# Slow sinusoidal tilt movements demonstrate the contribution to orthostatic tolerance of cerebrospinal fluid movement to and from the spinal dural space

**DOI:** 10.14814/phy2.14001

**Published:** 2019-02-27

**Authors:** Wim J. Stok, John M. Karemaker, Janneke Berecki‐Gisolf, Rogier V. Immink, Johannes J. van Lieshout

**Affiliations:** ^1^ Department of Medical Biology Section Systems Physiology Amsterdam UMC Location AMC University of Amsterdam Amsterdam The Netherlands; ^2^ Department of Medical Biology Laboratory for Clinical Cardiovascular Physiology Amsterdam UMC Location AMC University of Amsterdam Amsterdam The Netherlands; ^3^ Department of Anesthesiology Amsterdam UMC Location AMC University of Amsterdam Amsterdam The Netherlands; ^4^ Department of Internal Medicine Amsterdam UMC Location AMC University of Amsterdam Amsterdam The Netherlands; ^5^ Present address: Monash University Accident Research Centre (Vic Injury Surveillance Unit) Monash University Clayton Campus Clayton Victoria Australia

**Keywords:** Body position, cerebrospinal fluid, cerebrovascular autoregulation, computer model, orthostasis

## Abstract

Standing up elicits a host of cardiovascular changes which all affect the cerebral circulation. Lowered mean arterial blood pressure (ABP) at brain level, change in the cerebral venous outflow path, lowered end‐tidal P_CO__2_ (P_ET_CO_2_), and intracranial pressure (ICP) modify cerebral blood flow (CBF). The question we undertook to answer is whether gravity‐induced blood pressure (BP) changes are compensated in CBF with the same dynamics as are spontaneous or induced ABP changes in a stable position. Twenty‐two healthy subjects (18/4 m/f, 40 ± 8 years) were subjected to 30° and 70° head‐up tilt (HUT) and sinusoidal tilts (SinTilt, 0°↨60° around 30° at 2.5–10 tilts/min). Additionally, at those three tilt levels, they performed paced breathing at 6–15 breaths/min to induce larger than spontaneous cardiovascular oscillations. We measured continuous finger BP and cerebral blood flow velocity (CBFv) in the middle cerebral artery by transcranial Doppler to compute transfer functions (TFs) from ABP‐ to CBFv oscillations. SinTilt induces the largest ABP oscillations at brain level with CBFv gains strikingly lower than for paced breathing or spontaneous variations. This would imply better autoregulation for dynamic gravitational changes. We demonstrate in a mathematical model that this difference is explained by ICP changes due to movement of cerebrospinal fluid (CSF) into and out of the spinal dural sack. Dynamic cerebrovascular autoregulation seems insensitive to how BP oscillations originate if the effect of ICP is factored in. CSF‐movement in‐and‐out of the spinal dural space contributes importantly to orthostatic tolerance by its effect on cerebral perfusion pressure.

## Introduction

Standing up in the morning might be the most audacious deed of the day. It forces the circulation to adapt to the demands of gravity, most importantly to provide sufficient pressure for brain perfusion. Among the cardiovascular adaptations, the increased systemic vascular resistance (SVR) figures most prominently (Rowell 1993). In the face of a diminished venous return and, consequently, cardiac output, heart rate will increase by vagal withdrawal and sympathetic activation, compensating for a decreased stroke volume. After an initial 1‐ to 2‐min period of adaptation, arterial blood pressure (ABP) at heart level will have stabilized at a slightly higher level than before (Blomqvist and Lowell Stone 1983; Wieling and Karemaker 2013), but not sufficient to prevent a hydrostatic pressure drop at the level of the brain. Venous pressure inside the brain does not drop to the same extent; consequently, cerebral perfusion pressure is lowered (Rosner and Coley 1986; Qvarlander et al. 2013; Lawley et al. 2017). In these circumstances, obviously, autoregulation must come into play to uphold sufficient cerebral blood flow. This mechanism, as it has become known, has at least two separate components (Lassen 1959; Tiecks et al. 1995; Van Lieshout et al. 2003; Liu et al. 2013; Numan et al. 2014): a slowly adapting component in reaction to metabolic demands; and a fast, dynamic component, where the vessel walls react to changes in supply (Aaslid et al. 1989; Tiecks et al. 1995; Zhang et al. 1998; Panerai et al. 1999; Lucas et al. 2010; de Jong et al. 2017).

The dynamics of the fast component of autoregulation is generally defined by measurement of the transfer function (TF) from blood pressure to cerebral flow changes. For this measurement, the subject is kept in one position in relation to gravity, for example, lower body negative pressure (LBNP) (Birch et al. 2002; Brown et al. 2004; Hamner et al. 2004; Taylor et al. 2014; Smirl et al. 2015), standing up from sitting (van Beek et al. 2008; Oudegeest‐Sander et al. 2014), or squat‐stand maneuvers (Birch et al. 1995; Claassen et al. 2009; Barnes et al. 2017). No in‐depth studies have analyzed the TF when the gravity vector itself is being manipulated. A measurement under such circumstances is a more realistic reflection of a person's capacity to regulate cerebral blood flow, testing the ensemble of cardiovascular regulatory systems from baroreflex(‐es) to cerebrovascular autoregulation (Lucas et al. 2010), with all concurrent adaptations such as changes in intracranial pressure and venous outflow from the brain (Flexner and Weed 1933; Rosner and Coley 1986; Gisolf et al. 2004b; Qvarlander et al. 2013; Andresen et al. 2015; Lawley et al. 2017). This is what we set out to test in the present study, which is part of a larger investigation into predictive factors that define orthostatic tolerance in extreme circumstances like return from spaceflight (Buckey et al. 1996; Gisolf et al. 2005). Here, we used a computer‐driven tilt table, which has been designed to minimize vestibular excitation (Gisolf et al. 2004a).

Our test battery includes TF measurement from **(1)** spontaneous blood pressure to blood flow velocity oscillations in three body positions: supine, passive upright at 30° (0.5G), and tilted to 70° (almost 1 G), **(2)** by paced breathing in those three positions, thereby inducing larger than normal blood pressure oscillations, and **(3)** by a series of sinusoidal tilt frequencies from supine to almost upright around the 30° position.

We hypothesized that (1) the TF's from blood pressure to cerebral blood flow, measured in the three stable body positions, are similar, possibly differing by a scale factor due to the prevailing contractile state of the vasculature and concomitant changes in end‐tidal pCO_2_, and (2) the TF obtained by sinusoidal tilt is substantially different (in terms of gain and phase) due to the concurrent dynamic changes in hydrostatics of the venous and cerebrospinal fluid columns. Furthermore, we hypothesized that in the TF from sinusoidal tilt the lowest frequencies might deviate, since their time course would allow superimposed changes in activity of the autonomic nervous system and/or of pCO_2_ levels.

Finally, in view of the clinical use of quantifying the TF from blood pressure to cerebral blood flow, we compared the various measures (gain, phase) and ways of measuring them for stability and reproducibility.

## Methods

### Subjects

In total, 22 healthy subjects (four female) aged 40 ± 8 years, height 177 ± 9 cm, and weight 72 ± 11 kg participated in the study. Of the 22, seven were NASA astronauts destined for a shuttle flight and five ESA cosmonauts who would fly in the Soyuz capsule to the ISS for a short stay in Space. All participants were nonsmokers and were free of known cardiovascular and cerebrovascular disorders. Of the 22 subjects, eight underwent the whole protocol a second time after 12 months, enabling us to test the reproducibility over time of the various measurements.

The procedures were explained in detail, in written form and orally, before each subject signed the informed consent form. The protocol was approved by the Medical Ethics Committee of the Academic Medical Center, University of Amsterdam and, where appropriate, by the NASA and/or ESA medical review boards.

### Procedures

**Figure 1:**
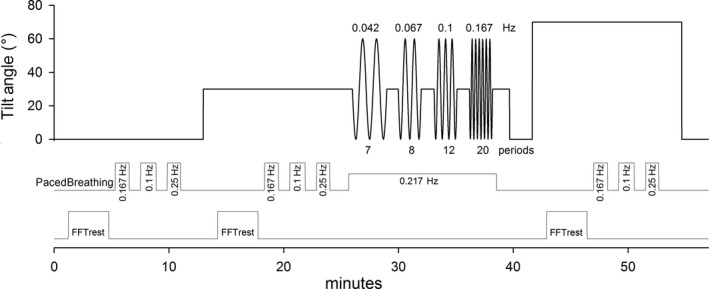
Schematic protocol and timing of tests. Upper panel: tilt angle, starting in the supine position, followed by 30° HUT, 4 periods of sinusoidal tilt (SinTilt) between 0° and 60°, and a period of 70° HUT. Middle panel: timings of the three paced breathing periods in each of the three fixed body positions plus the paced respiration during the SinTilt period. Lower panel: data parts where resting cerebral autoregulation was calculated.

All experiments were conducted in our laboratory at the Academic Medical Center location of the University of Amsterdam. Subjects were in the postprandial state after a light meal and had refrained from alcohol and caffeine for more than 12 h. The room was quiet and dimly lit at a temperature around 22°C. The subject was lying on the tilt table for the whole protocol, supported by a footboard, which was conveniently angled to 98° rather than 90° to prevent overstretching the calf muscles or forcing the ankle in an uncomfortable position. The procedures started with a few test tilts and breathing procedures to familiarize the test subject with the tilt movement and paced breathing. Figure 1 gives a schematic overview of the various tests.

The first series of tests was in the supine position: 5‐min resting, then *paced breathing (PB)* at 10, 6, and 15 per min, in that order, each for 1 min plus two extra breaths. Pacing was by audio cue: tone going up for inspiration, going down for expiration (40/60) followed by a short expiratory pause; the subject was instructed to only adhere to the cue for inspiration, and not to hyper‐ or hypoventilate, by keeping a LED bar indicating end‐tidal pCO_2_ (P_ET_CO_2_) within his/her normal limits.

The second series was in the 30° head‐up tilted (HUT) position, 5‐min resting recording, then PB, followed by sinusoidal tilts (SinTilt) around 30° at 2.5, 4, 6, and 10 per min, moving between 0° and +60°, each for 2 min, followed by 1‐min rest. To avoid synchronization of respiration to the tilt maneuvers, breathing was paced by audio cue at 13 per min; this continued for the duration of the tilt and resting maneuvers. The tilt angle plotted in time describes a sine between 0° and 60° at the given frequencies. A version of the SinTilt‐protocol has been used in an earlier publication from this laboratory (Bronzwaer et al. 2014).

The third set started with a tilt to 70° where the subject remained for 5 min. Then, PB was performed and, finally, a tilt down to supine.

### Data acquisition and preprocessing

Continuous noninvasive ABP was measured at the finger by a Finometer™ (TNO, BMI, Amsterdam, the Netherlands). The finger was held at the estimated level of the aortic valve throughout the procedures, the hand supported by a sling. Cerebral blood flow velocity (CBFv) was measured in the middle cerebral artery (MCA) by transcranial Doppler (TCD) (DWL, Germany). The probe was immobilized by a head band. The distance between aortic valve level and TCD probe was measured to calculate hydrostatic pressure‐corrected ABP at the brain level from the tilt angle: ABP_brain_. The electrocardiogram (ECG) was monitored (Hewlett Packard monitor 78345A). Carbon dioxide was sampled continuously from the nose and measured using a capnometer (Hewlett Packard Airway Adapter 1436A, coupled to the same monitor).

Finger blood pressure, the envelope of the TCD spectral curve, ECG, expiratory CO_2_, and the tilt angle were analog‐to‐digital converted at 100 Hz. Offline, beat‐to‐beat values of systolic, diastolic, and mean blood pressure, as well as the corresponding systolic, diastolic, and mean CBFv values were calculated, after low‐pass filtering the raw CBFv signal to a frequency of 15 Hz. The mean ABP (MAP) and CBFv (mCBFv) were computed as the integral over one beat. During a posture change, hydrostatic arterial pressure at the brain level will change by an amount ρgh_*_sin(*α*), where *ρ* is the density of blood, *g* the gravitational acceleration, *h* the vertical distance to the position of the heart, and *α* denotes the angle of tilt. The distance *h* was measured individually. To compensate for differences in travel time of the pressure wave from the heart to the brain and from the heart to the finger, the finger pressure wave was shifted 60 ms back in time, and then the continuously computed hydrostatic pressure difference was subtracted to derive ABP_brain_. P_ET_CO_2_ was derived from the CO_2_ in‐ and expiration curve.

Data segments of episodes of baseline rest, paced breathing, and tilting at each particular frequency were selected. At each tilt position, 3.5 min of recorded rest was used for spectral analysis. Ectopic beats were replaced by interpolation.

### Computation of TF's and spectral analysis

Two methods of transfer function analysis were applied: *for the rest periods* standard Fourier Transform was used of beat‐to‐beat MAP_brain_ and mCBFv. For this, the 3.5 min of data were spline interpolated and resampled at 4 Hz; the resulting 840 points data segment was linearly detrended and Hanning‐windowed. Power spectra were then computed by a discrete Fourier Transform (Matlab^®^ FFT routine). Resulting spectra were smoothed by a 7‐point triangular window. Linearity of the system and reliability of the transfer function estimation were evaluated by the coherence. Gain and phase relations for spontaneous oscillations were only included in further analysis for parts of the spectra with sufficiently high levels of coherence (i.e., >0.46 given the degrees of freedom and the chosen 95% confidence level). To account for intersubject variability in mCBFv, the TF gain was normalized by dividing the transfer function gain (cm/sec per mmHg) of a data segment by its average mCBFv (cm/sec), expressing it as %/mmHg. Phase is defined as positive where mCBFv leads MAP_brain_. The periods of paced breathing were introduced to force sufficient variability at a number of set frequencies to better define the cerebrovascular transfer function. Since paced breathing has a tendency to induce changes in P_ET_CO_2_, we kept the periods as short as feasible, while on the other hand still getting sufficient data for a good spectral resolution.

In spite of our precautions, P_ET_CO_2_ was not exactly constant during the *forced oscillations (PB and SinTilt)*; therefore, gain and phase relations were computed for those cases by fitting an autoregressive (ARX) model with MAP_brain_ as input and P_ET_CO_2_ as extra (exogenous) input (Ljung 1987; Liu and Allen 2002; Liu et al. 2003). For SinTilt, MAP_brain_ was replaced, at a later stage, by cerebral perfusion pressure (CPP = MAP_brain_ − ICP), where ICP changes during tilt were estimated from the beat‐to‐beat estimated critical closing pressure using the method of “first harmonics” extrapolation of the pressure and flow signal (Panerai 2003). In view of the noisiness of this signal, we fitted a model‐based descriptive curve to it. As explained in the Appendix for this purpose we adapted Ursino and Lodi's model of cerebrovascular autoregulation (Ursino and Lodi 1998) to include the movement of cerebrospinal fluid into and out of the spinal dural sack. The period of the tilt maneuvers at the four different frequencies (total ~12 min) and (separately) that of the three paced breathing frequencies (~7 min) were used in toto, including the in‐between resting periods of one minute. Gain and phase at the SinTilt and paced breathing frequencies were extracted from the derived TF's and compared to the data derived at rest as described above.

### Statistics

Data are presented as means ± SD. Differences between ARX gain and phase during the various stimuli were tested using the paired *t*‐test after checking for normal distribution. A Wilcoxon signed‐rank test was used for comparing not‐normally distributed data. Values for the intraclass correlation coefficients (ICC) were calculated from ANOVA models using SPSS Statistics V24 and the method described by Shrout and Fleiss (Shrout and Fleiss 1979). A significance level of *P* ≤ 0.05 was used throughout.

## Results

*Steady‐state values* for the main cardiovascular parameters in the three body positions (0°, +30°, and +70°) are presented in Table 1. Blood pressure at heart level increases only when going to +70°; at +30°, it is already significantly decreased at brain level. mCBFv decreases going from 0° to +30° and further to +70°, at the same time P_ET_CO_2_ is decreasing. CO decreases at +30° and remains unaltered at +70°, and SVR increases at +30° and still further at +70°.

**Table 1:** Hemodynamic and ventilatory subject parameters at three body positions

	Supine Rest	30° HUT Rest	70° HUT Rest
HR, beats/min	62 ± 12	67 ± 12[Link]	80 ± 11[Link] ^,^ [Link]
MAP, mmHg	77 ± 7	77 ± 8	87 ± 10[Link] ^,^ [Link]
MAP_brain_, mmHg	77 ± 7	62 ± 8 [Link]	60 ± 10[Link]
mCBFv, cm/sec	61 ± 15	56 ± 16[Link]	52. ± 14[Link] ^,^ [Link]
P_ET_CO_2_, mmHg	38 ± 3	36 ± 3[Link]	34 ± 4[Link] ^,^ [Link]
CO, L/min	4.3 ± 1	3.6 ± 0.7[Link]	3.5 ± 0.8[Link]
SVR, L/min per mmHg	1.14 ± 0.22	1.35 ± 0.25[Link]	1.57 ± 0.39[Link] ^,^ [Link]

Averaged values (*n* = 22) of the parameters over the resting periods of 3.5 min used for the calculation of the transfer functions between spontaneous oscillations of MAP_brain_ and mCBFv by FFT(^1^ indicates significant difference between HUT30° and Supine, ^2^ indicates significant difference between HUT30° and HUT70°, and ^3^indicates significant difference between HUT0° and HUT70°,). HR, Heart rate; MAP, mean arterial pressure; MAP_brain_, MAP at the brain level; mCBFv, mean cerebral blood flow; P_ET_CO_2_, end‐tidal pCO_2_; CO, cardiac output (by pulse contour from the Finometer device), SVR, systemic vascular resistance (Finometer). Values are mean ± SD.

### Dynamic values: Spontaneous variability, paced breathing, and sinusoidal tilt movements

**Figure 2:**
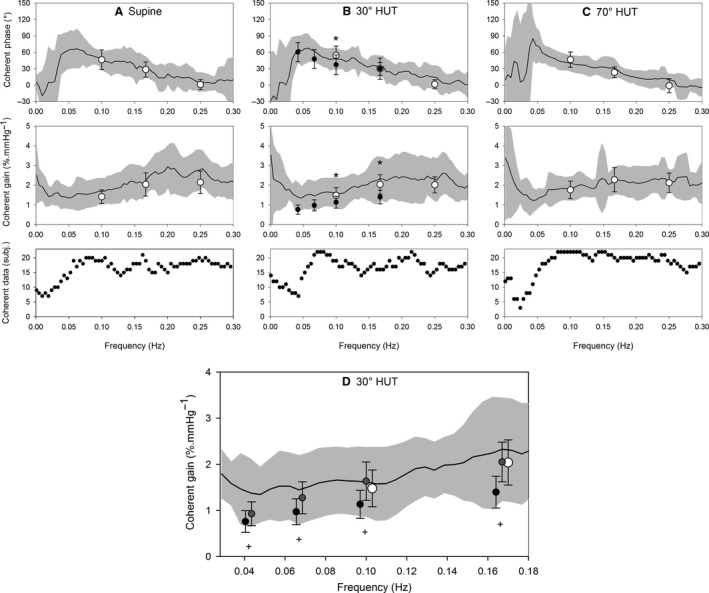
Transfer functions from MAP_brain_ to mCBFv in three positions (A: supine, B: 30° HUT, and C: 70° HUT) during rest (drawn black line: averaged cross‐spectral results where individual coherence ≥ 0.5, gray area indicates ± SD), paced breathing (PB, open circles ± SD, including all subjects and using an ARX model with MAP_brain_ and P_ET_CO_2_ as input and mCBFv as model output) and sinusoidal tilt (SinTilt, closed circles, including all subjects and an ARX model with MAP_brain_ and P_ET_CO_2_ as input and mCBFv as model output, but no compensation for changes in ICP). * indicates significant (*P* ≤ 0.05) difference between PB and SinTilt. D: TF gain at 30° HUT: black line and gray area, open and black closed circles as in fig 2B. Dark gray circles: gain during SInTilt after correction for estimated changes in intracranial pressure (ICP). + indicates significant difference (*P* ≤ 0.05) from values without correction.

**Figure A5:**
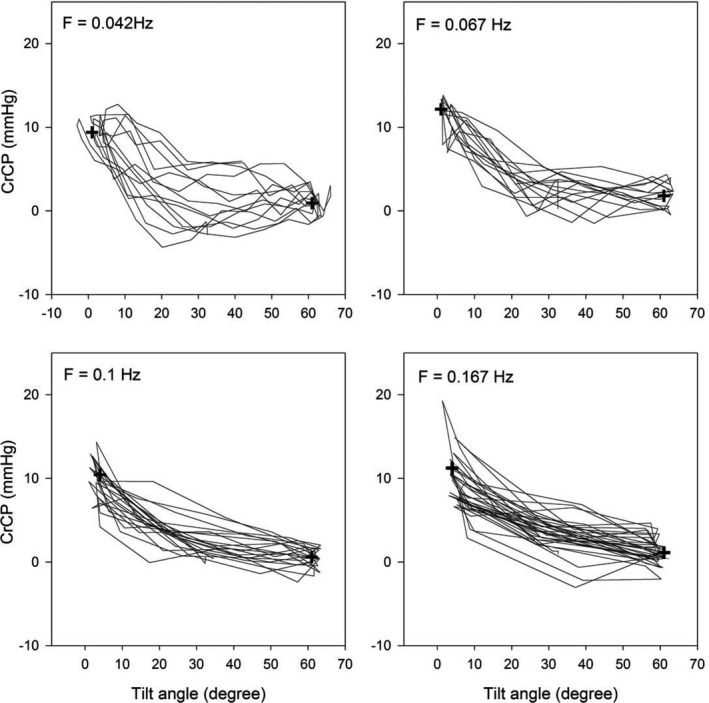
Calculated critical closing pressure (CrCP) versus tilt angle in one subject at four different sinusoidal tilt frequencies. Thin lines: one second resampled values from the original beat‐to‐beat data. Plus signs: averaged values at 0 and 60 degrees; these were used to calibrate the model‐derived relation of intra‐cranial pressure versus tilt angle.

Figure 4 shows an example of the measured parameters during the session in +30° tilted position, first 5 min resting recording, followed by 8 min for paced breathing at the three set frequencies, and finally, 13 min sinusoidal tilt at four frequencies. Note the large oscillation in CBF_v_ during the SinTilt maneuvers. Figure 2A–C give the averaged results for the transfer functions from MAP_brain_ to mCBFv in the three positions: supine, 30° HUT tilt, and 70° HUT tilt. The solid black line represents the overall averaged cross‐spectral results for those parts of the 3*22 spectra where coherence between MAP_brain_ and mCBFv was statistically significant. The open symbols are the results of the paced breathing and the closed symbols those of the SinTilt maneuvers. Striking is the similarity between the full spectral curves and how well the paced breathing data fit in. In the region from 0.05 Hz to 0.25 Hz, the curves behave as expected for a high‐pass dynamic autoregulation system (Tiecks et al. 1995; Zhang et al. 1998): decreasing phase advance of mCBFv to MAP_brain_ and increasing gain (i.e., more effect of pressure oscillations on flow). However, the SinTilt data do not fit this picture: the same phase behavior, but about two‐thirds of the gain. This would imply that the cerebral autoregulation (CAR) is somehow better able to filter out BP oscillations due to changes in gravitational load in the chosen frequency range. It was not explained by concurrent changes in P_ET_CO_2_ (cf. Fig. 4), since those had already been taken into account in Figure 2 (cf. Methods section). We reasoned that, conceivably, changes in ICP concurrent with the tilt maneuvers might play a role as well. Since a beat‐to‐beat extrapolation of ICP, or rather critical closing pressure (CrCP) (Panerai 2003), resulted in a noisy signal (cf. Appendix, Fig. A5), we looked for a mathematical way to get a smooth estimate. Therefore, we adapted Ursino and Lodi's (Ursino and Lodi 1998) model for CAR. This predicts the relation between pressure and flow by modeling the effects of autoregulation, ICP, and instantaneous pCO_2_ levels on cerebral vessel diameters. First, we had to adapt the model to meet the frequency requirements for the present case (detailed in the Appendix 1). Second, the movement of cerebrospinal fluid (CSF) between the cerebral and spinal compartments was considered to cause ICP changes during a posture change. Incorporation of the ICP variations into the ARX model brought the SinTilt data (both for gain and phase) more or less in line with the other measurements as shown in Figure 2D, numerically in Table 2.

**Table 2:** Transfer function MAP_brain_ – mCBFv

Frequency (Hz)	0.042	0.067	0.1	0.167	0.25
Stimulus/method					
Phase					
SinTilt: ARX	60.3 ± 17.7	47.8 ± 17.3	37.3 ± 18.0[Link]	30.0 ± 19.5	
ARX_ICP_	62.0 ± 17.6	51.5 ± 14.7	40.6 ± 16.6[Link]	23.8 ± 18.2	
PB: ARX			54.3 ± 17.2	29.8 ± 11.8	1.5 ± 8.3
Gain (% per mmHg)					
SinTilt: ARX	0.76 ± 0.23[Link]	0.97 ± 0.28[Link]	1.13 ± 0.30[Link] ^,^ [Link]	1.40 ± 0.35[Link] ^,^ [Link]	
ARX_ICP_	0.93 ± 0.26	1.27 ± 0.35	1.63 ± 0.42	2.05 ± 0.43	
PB: ARX			1.48 ± 0.40	2.04 ± 0.49	2.01 ± 0.41

Values are mean ± SD. Measured pressure (MAP_brain_) to mCBFv transfer function phase and gain (% per mmHg) in 22 subjects during sinusoidal tilt and paced breathing (PB) at 30° HUT with (ARX_ICP_) and without (ARX) correction for estimated intracranial pressure changes during tilt. (^1^indicates significant difference between ARX_ICP_ and ARX during tilt at the same tilt frequency, ^2^indicates significant difference between ARX and PB, and ^3^indicates significant difference between ARX_ICP_ and PB).

### Interindividual variability and reproducibility of gain and phase measurements

**Figure 3:**
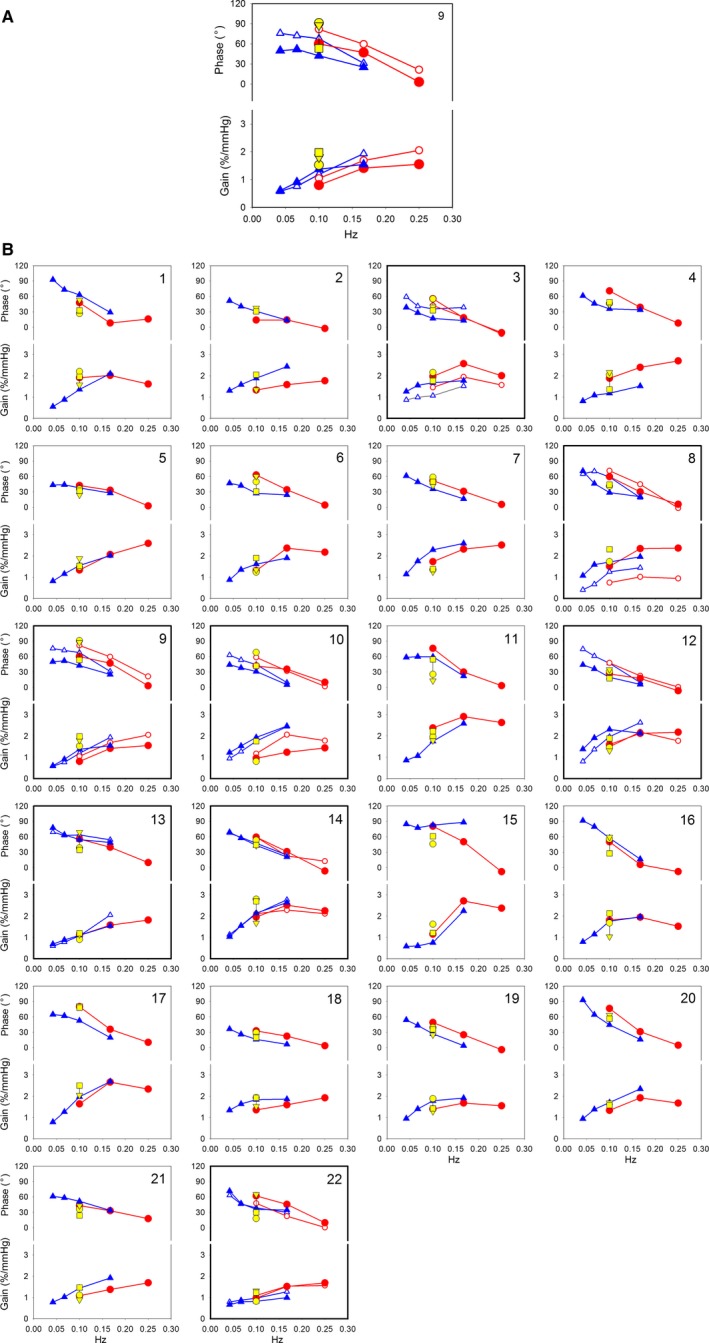
(A) Bode plots in subject 9 (as an example) in 30° head‐up tilted position with first and second measurement. SinTilt: blue diamonds at 0.042, 0.067, 0.1, and 0.167 Hz. Paced breathing: red circles at 0.1, 0.167, and 0.25 Hz. First and second measurements: closed and open markers, respectively. Additional yellow markers at 0.1 Hz: phase and gain during periods of rest in three body positions during the first measurement: supine (circle), 30° HUT (triangle), and 70° HUT (square). (B) Composite figure of phase and gain for all 22 subjects in 30° head‐up tilted position. Individual Bode plots of all 22 subjects of whom eight participated twice (bold axes).

We have demonstrated three methods to define the dynamic autoregulatory capacity of a person's cerebral circulation. Which one is best to apply? This “best” method must fulfill at least two requirements: (1) it should give reproducible results over time and (2) it can be applied without much patient involvement. To make a good comparison for the three ways to define the transfer from ABP_brain_ to CBFv, we have reduced the individual curves underlying Figure 2: For each subject, we only present the results for gain and phase at 0.1 Hz as derived from the spontaneous variability in supine, +30° and +70° HUT, respectively (Claassen et al. 2016) and the data points at the three frequencies PB and four frequencies SinTilt as measured at +30° HUT. The results, corrected for changes in P_ET_CO_2_ and ICP are summarized in Figure 3. Figure 3A shows an enlargement of one the individual summary curves for demonstration purposes, Figure 3B1–22 has the results for all 22 subjects. Whichever way one looks at it, these data show substantial inter‐ and intrasubject variability, as has been observed in many earlier studies (Birch et al. 2002; Panerai et al. 2003; Smirl et al. 2015).

### Reproducibility

Out of the 22 subjects, eight have participated in the whole procedure a second time, around 1 year after the first measurement. We have marked these eight in Figure 3 by heavy boxes, showing the results of both the first and second session. Factoring in these repeat measurements, we were able to compute the intraclass consistency and reproducibility for the three measurement techniques, shown in Table 3. This shows that only the SinTilt maneuvers give reproducible results, except at the lowest frequency of 0.042 Hz. Paced breathing performed poorly in terms of reproducibility, only a frequency of 0.167 Hz (10 per min) gave reproducible results. However, for the purpose of determining pressure to cerebrovascular blood flow transfer function, this frequency is too high (i.e., the changes are too rapid). From the spontaneous oscillations, only the 70° HUT results at 0.1 Hz were reproducible.

## Discussion

In a recent “crosstalk” discussion in the *Journal of Physiology*, Drs. Simpson and Claassen defended the view that dynamic CAR should be quantified using induced blood pressure oscillations (Simpson and Claassen 2018a,b), a view which was strongly contested by Drs. Tzeng and Panerai, pointing at the possibility that nonlinearities are introduced if larger than the spontaneous oscillations around baseline values are induced (Tzeng and Panerai 2018a,b). The present study used two methods of inducing increased blood pressure oscillations: sinusoidal tilt and paced breathing, while at the same time comparing the results to those from analysis of spontaneous oscillations, concurrent in ABP and CBFv.

**Figure 4:**
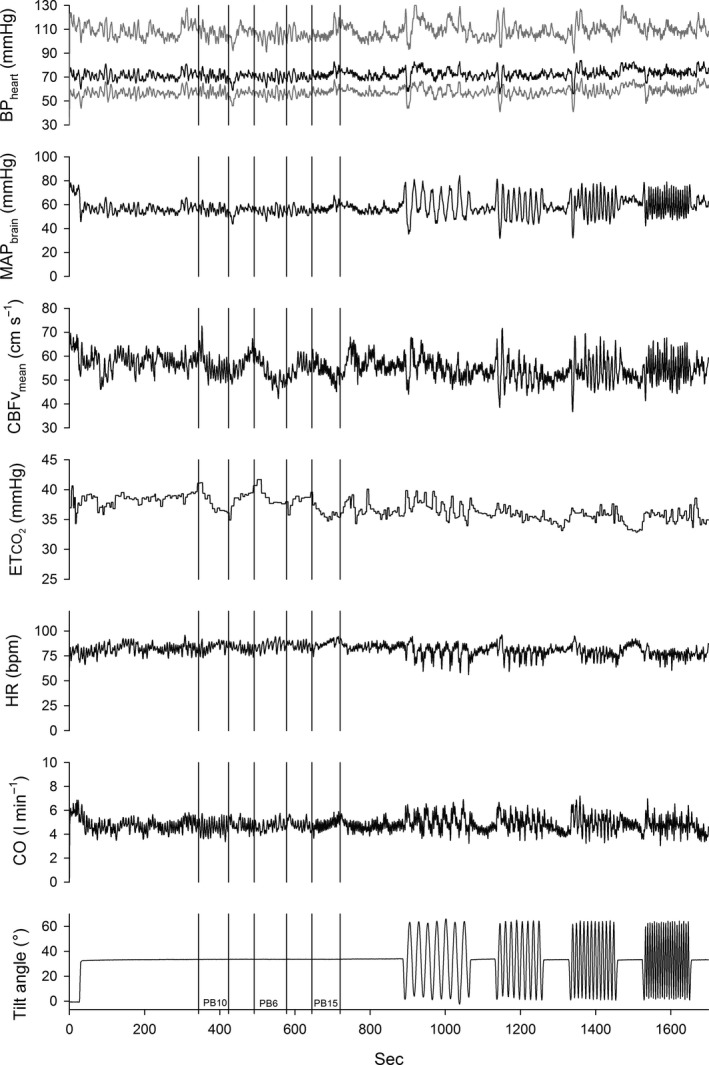
Example of session in the 30° HUT position: 5 min of rest recording, followed by paced breathing at three frequencies, and sinusoidal tilt at four frequencies. During the latter maneuvers, respiration was paced at 13 breaths/min. In the upper panel systolic, diastolic (gray) and mean pressures (black) are displayed.

Not many studies have used dynamic changes in gravitational load to study the effect on cerebral blood flow. In 2001, Hughson et al. (2001) applied square wave up and down movement of a tilt table: 10‐sec HUT to 45°, 10‐sec supine. This results in a basic period of 20 sec, slightly shorter than our lowest sinusoidal tilt frequency of 24 sec. The Fourier transform of a square wave maneuver consist of many higher order harmonics, in addition to the average value of around 22.5° HUT which was applied. In our experimental setup, this was prevented by tilting at precisely defined sinusoidal frequencies, from 0° to 60° starting at a baseline of 0.5 G or 30°. Furthermore, we asked the test subjects to pace their breathing to a constant frequency which was chosen so as not to interfere with any of the tilt frequencies. In addition, visual feedback was provided on end‐tidal pCO_2_, in the expectation that this would stabilize its level. In spite of all these precautions, pCO_2_ was not constant (cf. Fig. 4). Since CO_2_ is known to be a potent vasodilating factor for cerebral blood flow (Immink et al. 2014), we had to compensate for this in the comparison between the various interventions. We chose to “blend” the three paced breathing and (separately) the four SinTilt frequencies in an ARX model, where the CO_2_ level was introduced as extra input variable. Even then, the three methods of defining the TF: spontaneous oscillations, paced breathing and sinusoidal tilt would not agree. Only by considering the shift of cerebrospinal fluid in and out of the spinal compartment under gravitational loading could the three be brought “in line” except for the lowest tilting frequency (0.04 Hz, 24 sec period) cf. Figure 2D. As explained in more detail in the Appendix  intracranial pressure decreases as CSF flows away from the brain cavity under the influence of gravity when one is tilted to a more upright posture. This will counteract the decreased arterial pressure, since it leads to an increase in cerebral perfusion pressure. Consequently, it increases the pressure‐head while at the same time decreasing the amplitude of the gravity‐induced pressure oscillation. In the calculation of the gain, one only looks at the effect of the oscillatory part; a reduction in the denominator (blood pressure variations) will then increase the resultant gain. This is exactly what the model explains quantitatively and it is sufficient to bring the SinTilt gain data in line with the other measurements.

By the use of oscillatory LBNP, Hamner et al. (2004) and Taylor et al. (2014) found that the coherence between induced blood pressure oscillations and observed CBFv oscillations was decreasing at frequencies below 0.07 Hz. This phenomenon remained unexplained. Likewise, various authors have used maneuvers to enhance blood pressure oscillations in the very low‐frequency range, by repeated squat‐stand maneuvers (Birch et al. 1995; Claassen et al. 2009; Barnes et al. 2017) or sit‐stand (van Beek et al. 2008; Oudegeest‐Sander et al. 2014). All observed decreasing amplitude of CBFv oscillations with decreasing frequencies, even when coherence remained high. Since the direction of the gravitational vector did not change in the LBNP‐ or in the squat‐/sit‐stand‐maneuvers, the authors did not check for indications of concurrent changes in ICP. In our view, this is an omission: if the pressure in the abdominal cavity changes, so will the pressure in the spinal compartment. We expect that part of the responses in those experiments are due to concurrent changes in ICP, thereby invalidating the assumption that (changes in) blood pressure equal (changes in) cerebral perfusion pressure.

**Table 3:** Intraclass correlation coefficient (ICC) of gain and phase for first and second visit in eight subjects

Maneuver (*n*)	Gain						Phase					
	‐*CO*_*2*_			*+CO*_*2*_			*‐CO*_*2*_			*+CO*_*2*_		
	ICC	Lower bound	Upper bound	ICC	Lower bound	Upper bound	ICC	Lower bound	Upper bound	ICC	Lower bound	Upper bound
SinTilt (Hz)(8)												
0.042	0.288	−0.250	0.774	0.407	−0.405	0.848	0	−0.524	0.629	0.318	−0.490	0.816
0.067	0.453*	−0.072	0.842	0.717*	0.105	0.936	0.348	−0.093	0.783	0.587	−0.132	0.901
0.1	0.804*	0.362	0.956	0.950*	0.791	0.990	0.333*	−0.024	0.757	0.679*	0.093	0.924
0.167	0.778*	0.277	0.950	0.751*	0.175	0.944	0.788*	0.293	0.953	0.815*	0.345	0.960
PB (Hz) (7)												
0.1	0.616	−0.073	0.909	0.529	−0.241	0.885	0.380	−0.290	0.828	0.203	−0.681	0.780
0.167	0.042	−0.751	0.707	0	−0.984	0.597	0.738*	0.163	0.941	0.533	−0.236	0.887
0.25	0	−0.870	0.329	0	−1.307	0.409	0.275	−0.533	0.800	0	−0.902	0.649
Rest 0.1 Hz												
Supine (8)	0.424	−0.325	0.836	0.585*	−0.027	0.886	0.320	−0.444	0.797	0.222	−0.580	0.761
HUT 30° (7)	0.145	−0.746	0.785	0.044	−0.904	0.755	0.142	−0.772	0.786	0	−1.037	0.659
HUT 70° (8)	0.641*	0.011	0.906	0.598*	−0.054	0.893	0.502*	−0.057	0.851	0.009	−0.574	0.625

Intraclass correlation coefficients (agreement) during sinusoidal tilt (eight subjects), paced breathing at 30° HUT (PB) (seven subjects) and resting recordings in three body positions (7–8 subjects) who participated a second time almost a year after the first. Values are calculated separately for gain and phase and for each of the stimulus frequencies during SinTilt, PB, and rest, respectively (*indicates *P* ≤ 0.05). Inclusion of the absolute P_ET_CO_2_ level during each stimulus as covariate in the ANOVA model increased ICC during SinTilt considerably. During PB only at 0.167 Hz and without P_ET_CO_2_ in the model, a significant ICC could be determined. In the resting recordings, only at HUT 70° significant ICC values were present in both gain and phase. Values of 0 indicate that no valid ANOVA model was found. (Lower‐ and upper bounds are the 95% confidence limits.)

In the aggregate, the TF's found in this study fit well to those reported in the literature (cf. Fig. 2); however, the individual curves in Figure 3 and the results after 1 year, as detailed in Table 3, demonstrate that there is more here than only the effect of blood pressure on CBFv, even more than the effects of CO_2_, since the ARX model used for the analysis took that into account. Possibly, we are here again looking at the effects of intraspinal pressure changes, if subjects are breathing more forcefully than was actually requested. In a recent study (Dreha‐Kulaczewski et al. 2018), respiration‐driven movement up and down the spinal and into the cerebral space has been observed. In our study, only indirect evidence was found for changes in intracranial pressure, by the extrapolation of arterial pulse wave and the concomitant CBFv‐pulse (cf. Appendix, Fig. A5)

This study was undertaken on the assumption that dynamic (passive) changes of body posture might shed more light on the individual capacity to autoregulate cerebral blood flow. The results of these studies were to help predict orthostatic intolerance after challenges like (short‐lasting) space flight. Part of this intolerance has been attributed (Buckey et al. 1996) to failing CAR, which was not measured at the time. None of the five cosmonauts in our study showed orthostatic intolerance after return from the ISS, although they would display extremely variable blood pressure after standing up in the morning, immediately after their return (Gisolf et al. 2005). The fate of seven Columbia astronauts belongs to the dramatic chapters of manned space flight, since their space shuttle perished on re‐entry.

In our analysis of the CAR data, we have tried to find clusters of responses, but the scatter within this small group of 22 healthy subjects would not allow a meaningful grouping of types of responses. Obviously, the variation in “normal” responses to the interventions which we applied can be very large without unmasking orthostatic problems in cerebral blood supply.

## Conclusions

We set out to test CAR during an orthostatic challenge and used dynamic tilt maneuvers as a novel method, expecting it to differ from more classical methods to define the transfer from ABP_brain_ to CBFv. Indeed, at first sight, CAR can dampen gravitationally induced oscillations better than spontaneous ABP oscillations or those provoked by paced breathing when the subject is in a stable position. This improvement is due to the concurrent changes in intracranial pressure, when CSF flows to and from the spinal dural space subject to the tilt maneuvers. With this effect taken into account, CAR seems insensitive to how it is tested, apart, maybe, from the slowest tilt frequency we used (0.042 Hz), where other factors may come into play.

Of the methods that were tested for their use in the measurement of CAR, we found that sinusoidal tilt, relatively, gave the best reproducible results. Moreover, this technique can be applied without extensive subject cooperation and it lends itself to investigating very low frequencies with a high degree of precision.

## Limitations

The study has a limited (22) number of mainly male participants (4/18, f/m). This is explained by the study design, where the group of astronauts/cosmonauts (2/10, f/m) was to be complemented with a control group of roughly the same size and composition. In spite of the precautions which we took to get “ideal” responses (CO_2_ feedback, fixed respiration), still the CO_2_ levels were not exactly constant during SinTilt, contributing to the spread in results.

As in all studies where TCD of one main cerebral artery is used to estimate cerebral blood flow, we have to assume that the various interventions did not influence vessel diameter to such an extent that it would invalidate our interpretation. Furthermore, the observed changes in P_ET_CO_2_ during tilt movements may not express the full extent of arterial CO_2_ (Immink et al. 2009).

In the present paper, we focused on the reactions of CBFv to changes in cerebral perfusion pressure, taking the blood pressure, as it were, for granted. The analysis of how blood pressure was maintained in the various tests is outside the scope of this paper and requires a study of its own. However, the sympathetic part of blood pressure control may have influenced the cerebral vessels (Zhang et al. 2002; Ainslie and Brassard 2014; Verbree et al. 2017) as it did the other systemic vessels. On the other hand, we have tried to limit the sympathetic activation in the SinTilt challenges as much as possible by our swift tilt table movements. All in all, we consider our SinTilt‐test the best option for dynamic testing of orthostatic cerebrovascular autoregulatory capacity.

## Conflict of Interest

None declared.

## Figures and Tables

**Figure A1:**
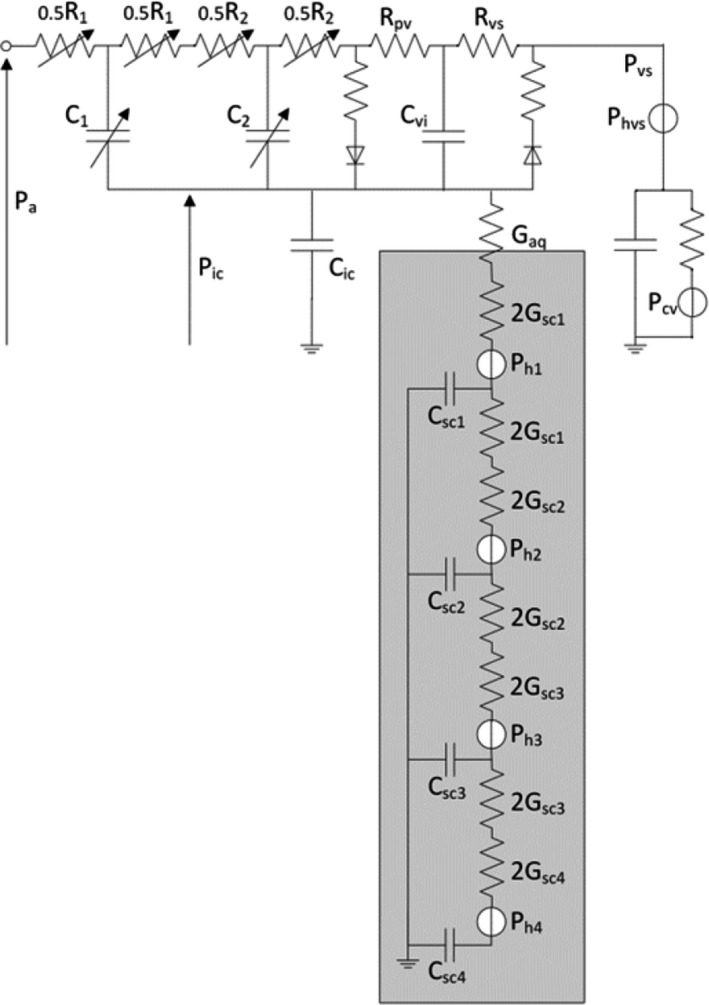
Model of cerebrovascular autoregulation adapted from Ursino (Ursino and Lodi 1998), showing two arterial compartment blocks with addition of a spinal cord part (grey area) in parallel with intracranial compliance *C*_ic_. Hydraulic conductance and compliance for each spinal segment are indicated by *G*_sc*j*_ and *C*_sc*j*_ respectively, where cs is spinal cord and j is the segment number. Hydrostatic pressure differences for the segments are represented by the pressure sources Ph_j_ and Ph_vs_. *G*_aq_ is upper cervical canal conductance for CSF. *P*_a_, *P*_ic_, *P*_vs_ and *P*_cv_ are model input arterial, intracranial, sinus venosus and central venous pressure respectively. Autoregulation is implemented by the variable conductance and compliance in the first and second arterial segment respectively.

**Figure A2:**
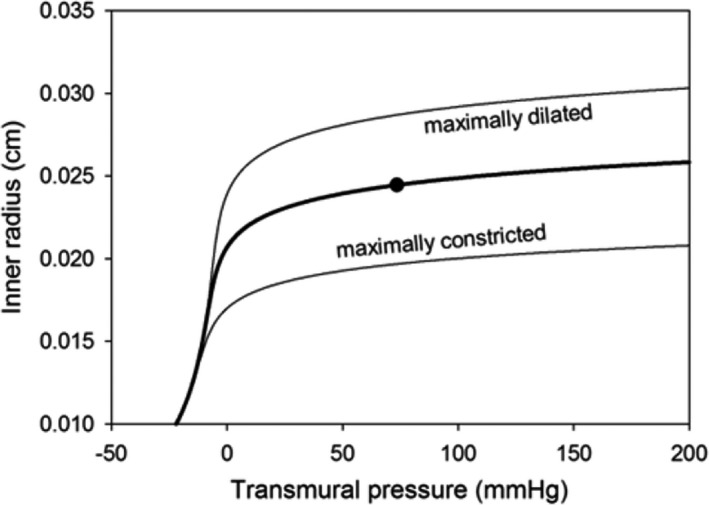
Pressure‐diameter relation in the basal (black line) and in the completely dilated or constricted condition in the first arterial model segment. Black dot: value at a segment transmural pressure of 76 mmHg.

**Figure A3:**
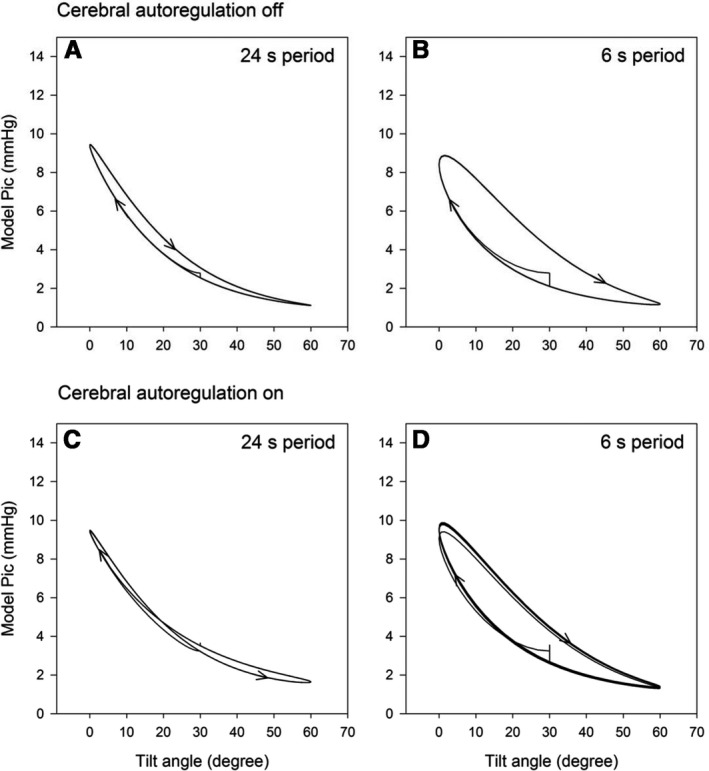
Model intra‐cranial pressure (MoP_ic_) versus tilt angle during the slowest (24 s period) and fastest (6 s period) sinusoidal tilt with cerebral autoregulation (Lucas et al. 2010) switched off (upper panels) and with CA set to dynamics according to ARI = 5 (lower panels).

**Figure A4:**
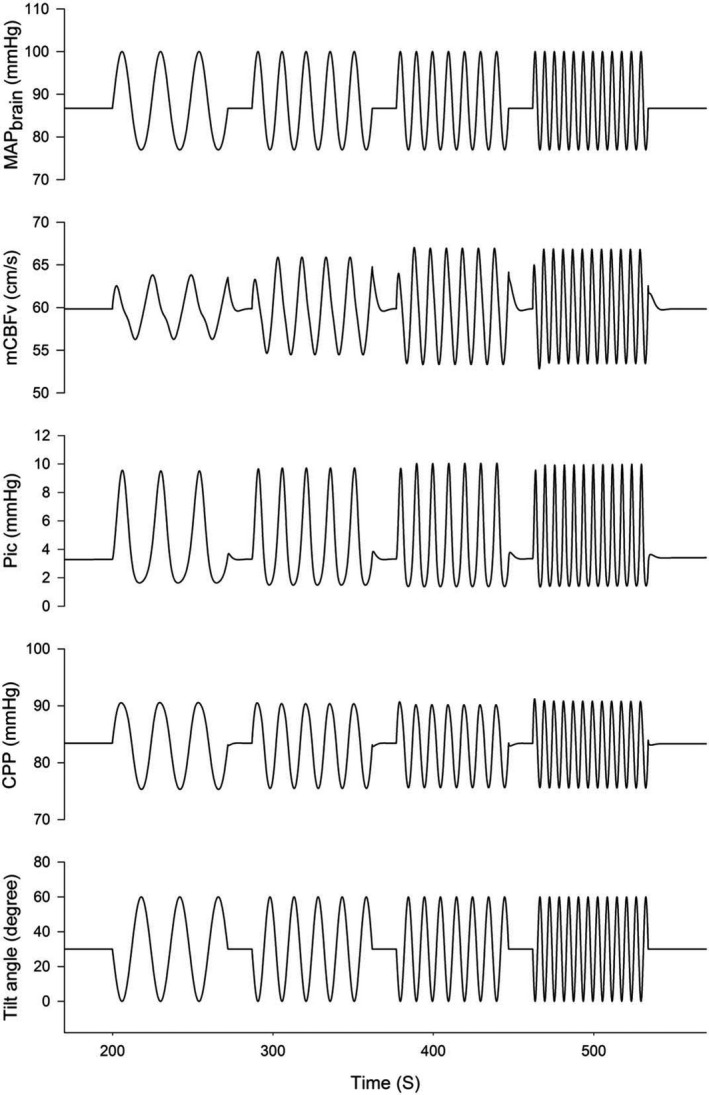
Model response for oscillatory tilt at four frequencies (0.042, 0.067, 0.1 and 0.167 Hz, lower trace). MAP_brain_: mean arterial pressure at brain level due to hydrostatic pressure changes, mCBFv: mean flow velocity in the middle cerebral artery, *P*ic: intracranial pressure, CPP: cerebral perfusion pressure. Model dynamics set according to an autoregulation index (ARI) of 5.

## Cerebrovascular model

To understand the cerebrovascular dynamics during posture changes we used Ursino and Lodi's model (Ursino and Lodi 1998), with some adaptations. In the first place we added a *spinal cord model* to simulate the displacement of CSF from the cranial to the spinal compartment and back. Furthermore, we had to adapt *autoregulatory dynamics* and the *elastic properties* of the cerebral vessels in the model to better approximate the fast dynamics as they were met in the present experiments. Figure A1 depicts the elements of the adapted model.

### Spinal cord model

The spinal cord compartment was modelled in parallel to the intracranial compliance as a series of Windkessel segments representing hydraulic resistance of the spinal CSF space (Elliot et al. 2011) and local compliance respectively (Fig. A1). We used four segments of equal length in series, each having a pressure source representing the hydrostatic pressure difference over the segment. The original total intracranial (ic) cerebrospinal compliance *C*_ic_ as given by Ursino was divided over the cerebral and spinal (sc) compartments with a 33% versus 67% contribution of the cranial and combined spinal compartments respectively (Lofgren and Zwetnow 1973; Magnaes 1989; Tain et al. 2011). The lumbar segment was considered to possess a higher compliance than the more cranial segments (Hogan et al. 1996; Linninger et al. 2009). Hydraulic conductance values G were taken from Elliot et al. 2011 and Ambarki et al. (2007) (Table A1).

The intracranial and 1‐4th spinal dural sac compliances, defining their capacity to store CSF‐volume (*C*_ic_ and *C*_sc,j_ *j *=* *1,..,4) were considered inversely related to local pressure (*P*_ic_ resp. *P*_sc,*j*_):

(A1) $$C=1/(k_{E}\cdot P)$$

where *k*_E_ is the local elastance coefficient and *P* is the sum of volumetric and hydrostatic pressure. Volume balance at each spinal segment is:

(A2) $$\begin{array}{cc} & C_{\mathrm{sc}}{}_{,j}\cdot \left(dP_{\mathrm{sc}}{}_{,j}/dt\right)=G_{\mathrm{sc}}{}_{,j}\cdot \left(P_{\mathrm{sc}}{}_{,(j-1)}-P_{\mathrm{sc}}{}_{,j}-{\text{Ph}}_{\mathrm{sc}}{}_{,j}\right)\\ & -G_{\mathrm{sc}}{}_{,(j-1)}\cdot \left(P_{\mathrm{sc}}{}_{,j}-P_{\mathrm{sc}}{}_{,(j+1)}-{\text{Ph}}_{\mathrm{sc}}{}_{,(j+1)}\right)\;\text{for}\;j=1,..,4 \end{array}$$

where *j* is the segment number, counting from the cranial side, Ph_sc_ is hydrostatic pressure and *G*_sc_ is segment conductance. For *j *=* *1, *P*_sc,(*j*‐1)_ = *P*_ic_ and *G*_sc,*j*_ includes the hydraulic conductance inside the upper cervical canal/foramen occipitale magnum. For *j* = 4 (last, lumbar segment), parameters with *j* + 1 suffix are zero. Hydrostatic pressure differences within the spinal cord segments are calculated as *ρgh*·sin(*α*), where *h* represents the distance between the segment location and the skull or between segments, *α* denotes the angle of tilt, *ρ* is the density of blood and *g* is the gravitational acceleration. The total spinal cord length was estimated to be 65 cm for an adult with a height of 180 cm as assumed in the simulations. A hydrostatic pressure source was added in the extracranial venous pathway to lower the venous sinus pressure in proportion to the tilt angle (Qvarlander et al. 2013). Thereby venous sinus pressure remained lower than model ICP (MoP_ic_)(16).

### Autoregulatory Dynamics

To have the model react to step changes of ABP with ARI‐like responses (Tiecks et al. 1995) we had to adapt the gains and time constants of the large and small arterial segment diameter regulation (Fig. A1, variable conductance and compliance parts). Compared to Ursino and Lodi's original model we used around 50% shorter time constants and, moreover, second order dynamics, the latter also used by (Diehl et al. 1995; Zhang et al. 1998). Adapted model parameters are given in Table A1.

### Vascular Diameter Regulation

The shape of the pressure‐diameter curve was chosen in line with the properties of muscular arteries under different levels of activation as found by Nichols and Edwards (2001) and Bank et al. (1995) (cf. Fig. A2). In the original model an activation factor M was introduced to represent the degree of smooth muscle contraction in the given arterial segment. *M* = 0 describes the basal, non‐activated condition, *M* = 1 maximally activated, *M* = −1 maximally dilated. We have followed this convention; however, this required some adaptations. The influence of the active muscular tension is represented here by a factor *fm* in the calculation of the elastic stress *σ*_e_ that shifts the elastic tension curve in dependence of Ursino's regulating activity *M*_*j*_ for the first and second arterial segment (*j*) respectively.

(A3) $$\sigma_{e}{}_{,j}=\sigma_{e0}{}_{,j}[\text{exp}(K_{\sigma ,j}\cdot \frac{r_{j}\;-{\;r}_{0,j}\;*\;fm_{j}}{{\;r}_{0,j}\;*\;fm_{j}}\;)-1]-\sigma_{\mathrm{coll}}{}_{,j}\;j=1,2\;\;\left(\text{coll}=\text{collapse}\right)$$

(A4) $$T_{e}{}_{,j}=\sigma_{e}{}_{,j}\cdot h_{j}\;j=1,2$$

where *r*_0_ is the inner radius of the unstressed wall and *σ*_e0,_ *K*_*σ*,*j*_ and *σ*_coll,*j*_ are constants, *T*_e_ is tension and *h* is wall thickness. Tension is negative before vessel collapse due to factor *σ*_coll._ Parameter *fm* is dependent on the activation factor M_*j*_ in such a way that in the basal condition of the model (*M*_*j*_ = 0, *fm = 1*) and in the fully dilated condition (M_*j*_ = ‐1) arterial diameters are the same as in the original model. In a polynomial adaptation to Ursino's model curves we set *fm* for the first segment to:

(A5) $$\begin{array}{c} fm=(\left(-0.0123*{M1_{1}}^{2}-0.1537*M1_{1}\right)*T_{\max0,1}/2.16)+1 \end{array}$$

and for the second segment:

(A6) $$fm=(\left(0.0174*{M1_{2}}^{4}-0.1396*{M1_{2}}^{3}+0.4332*M1_{2}^{2}-0.6971*M1_{2}\right)*T_{\max0,2}/1.473)+1$$

where M1_j_ = 1 + M_*j*_ and T_max0,*j*_ is a constant.

Model calculations were performed using Simulink^®^ (MATLAB^®^ 2007b) with Ode23 solver.

With these adaptations the model's transfer function (from input pressure to resultant cerebral flow‐velocity) can successfully be fitted to the responses described for different autoregulatory indices (ARI's) (Tiecks et al. 1995). To simulate the default behavior of the model during HUT, gain and time constant values were chosen such that the model would display a transfer function equivalent to an ARI value of 5 (Table A1). Values for *τ*_aut_ are about twice as small as in the original model. Other parameters different from the original are also presented in this table.

**Table A1:** Values of model parameters

Parameter values		
*τ*_aut,11_ = 2.174 sec		*τ*_aut,21_ = 10.434 sec
*τ*_aut,12_ = 3.199 sec		*τ*_aut,22_ = 12.388 sec
*G*_aut,1_ = 0.0094 mmHg^−1^		*G*_aut,2_ = 3.4742 mmHg^−1^
		
	*K*_MCA_ = 8	
	*k*_E_ = 0.2973 mL^−1^	
	*k*_E‐SC1‐3_ = 1.0529 mL^−1^	
	*k*_E‐SC4_ = 0.3475 mL^−1^	
	*G*_aq_ = 5 mmHg^−1^·sec^−1^·mL	
	*G*_sc_1‐3_ = 1.72 mmHg^−1^·sec^−1^·mL	
	*G*_sc_4_ = 5.16 mmHg^−1^·sec^−1^·mL	

Model parameters that are additional or changed in relation to the original study of Ursino ((59)). For *τ*_aut_ and *G*_aut_ the first index relates to the first and second arterial segment respectively. With the given values the model response is corresponding to an ARI of 5 in the supine position.

### Model simulation results

We tested the final model by subjecting it to the parameters and tilt frequencies of the SinTilt maneuvers. Starting at MAP as measured at heart level, arterial pressure at brain level was calculated taking the average hydrostatic distance of heart‐brain (MCA level) of our test subjects into account, i.e. 34 cm. The computed changes in model ICP (MoP_ic_) were then calculated. Figure A3 shows the relation MoP_ic_ versus tilt angle at the slowest and fastest tilt with autoregulation switched off and then on. At a tilt angle of 20° HUT MoP_ic_ is already halfway the final upright value (Fig. A3A and C). With model autoregulation switched off (Fig. A3A and B), hysteresis in the MoP_ic_ – tilt angle relation is present at all rates of tilting, gradually increasing with faster tilts. This hysteresis was related to a simple delay of 0.4 s. With intact autoregulation only at the fastest tilt period of 6 sec a clear hysteresis is present, while the relation was more or less 8 shaped (Fig. A3C) during the other tilt speeds. Besides these small differences the shape of the MoP_ic_ versus tilt angle relation was independent of the level of autoregulation. Because of the small value of the delay in relation to the average subject's heart interval (931 ± 166 msec) and the final sample rate for the ARX model of 1 Hz, no delay compensation was used in further analysis. A simulation run of all 4 tilt frequencies showing the most relevant parameters is presented in Figure A4. The relation tilt angle (*α*) ‐ MoP_ic_ from Figure A4 was used in the later data analysis as fitted by a 4th degree polynomial with parameters:

(A7) $$P_{\mathrm{ic}}=1.091e^{-7}\alpha^{4}-3.669e^{-5}\alpha^{3}+0.005002\alpha^{2}-0.3222\alpha +9.945$$

### Application to the actual recordings

To arrive at a reliable estimate of ICP during the SinTilt maneuvers, we estimated as a first step the CrCP using the first harmonics of the pressure and flow velocity waves (Aaslid et al. 2003; Michel et al. 1997; Panerai 2003). However, these values are too noisy and unstable to be used beat‐to‐beat as an estimate of ICP. Therefore, we only used the individual averages of the ‘turning points’ of these curves at 0° and 60° (Fig. A5). With these points as anchors we scaled formula (A7) to get a more stable estimate of dynamic ICP. These values were subtracted beat‐by‐beat from the MAP_br_ to arrive at CPP which was used as input signal for the ARX‐TF calculation.
